# *Helicobacter pylori*-Induced Chronic Gastritis and Assessing Risks for Gastric Cancer

**DOI:** 10.1155/2013/393015

**Published:** 2013-07-29

**Authors:** Gonzalo Carrasco, Alejandro H. Corvalan

**Affiliations:** ^1^Department of Pathology, Mount Sinai School of Medicine, 1425 Madison Ave, New York, NY 10029, USA; ^2^Centre for Translational Research in Oncology (CITO) and Department of Hematology and Oncology, Pontificia Universidad Catolica de Chile, Marcoleta 391, 8330074 Santiago, Chile

## Abstract

Chronic gastritis is an inflammation of the gastric mucosa and has multiple etiologies. Here we discuss the pathological alterations induced by *Helicobacter pylori* (HP) leading to chronic gastritis and the epigenetic bases underlying these changes. We review the histology of the normal gastric mucosa and overview the role of HP in the multistep cascade of GC. We attempt to define the role of the Operative Link for Gastritis Assessment (OLGA) staging system in assessing the risk of GC. The epigenetic bases of chronic gastritis, mainly DNA methylation, are presented through examples such as (i) the methylation of the promoter region of E-cadherin in HP-induced chronic gastritis and its reversion after HP eradication and (ii) the association of methylation of the promoter region of Reprimo, a p53-mediated cell cycle arrest gene, with aggressive HP strains in high risk areas for GC. In addition, we discuss the finding of RPRM as a circulating cell-free DNA, offering the opportunity for noninvasive risk assessment of GC. Finally, the integration of OLGA and tissue biomarkers, by systems pathology approach, suggests that severe atrophy has a greater risk for GC development if, in addition, overexpressed p73. This trial is registered with ClinicalTrials.gov NCT01774266.

## 1. Introduction

Since 1870, both human and veterinary pathologists have described bacterial infections based on the observation of tiny curved bacteria within gastric mucosa [1, 2]. However, these organisms were dismissed as irrelevant contaminants. In 1947, when gastroscopy was first being used, Schindler deemed gastritis as “one of the most debated diseases of the human body” and predicted that its significance would be discussed “for some time to come” [3]. Schindler himself claimed that the “bacteriological etiology of chronic gastritis has not been convincingly proved in a single case” [3]. In 1984, Marshall and Warren proposed that chronic “idiopathic” gastritis had a bacterial cause, that is, *Helicobacter pylori* [4]. Their hypothesis was met with great skepticism. However, within a few years, the association between *H. pylori* gastritis, peptic ulcer, and gastric cancer came to be acknowledged and ultimately accepted [4]. For the purpose of this paper, we will focus mainly on the cascade of events produced by *Helicobacter pylori* infection leading to chronic changes in the gastric mucosa and the risk assessment for the development of gastric cancer. In addition, we will explore the epigenetic bases that underlie the changes of chronic gastritis.

## 2. Normal Gastric Histology

In order to recognize pathologic tissue responses in gastritis, it is essential to know the spectrum of normal gastric mucosa histology patterns. Normal gastric mucosa is formed by the epithelial/glandular and lamina propria components. The epithelial component consists of the foveolar epithelium, which is formed by tall columnar mucous cells with basally situated nuclei and supranuclear collections of closely packed small mucus globules that discharge their content onto the surface, forming an adherent protective lubricant layer that lines the lumen. The glandular component changes depending on its location in the stomach.

**Figure 1:**
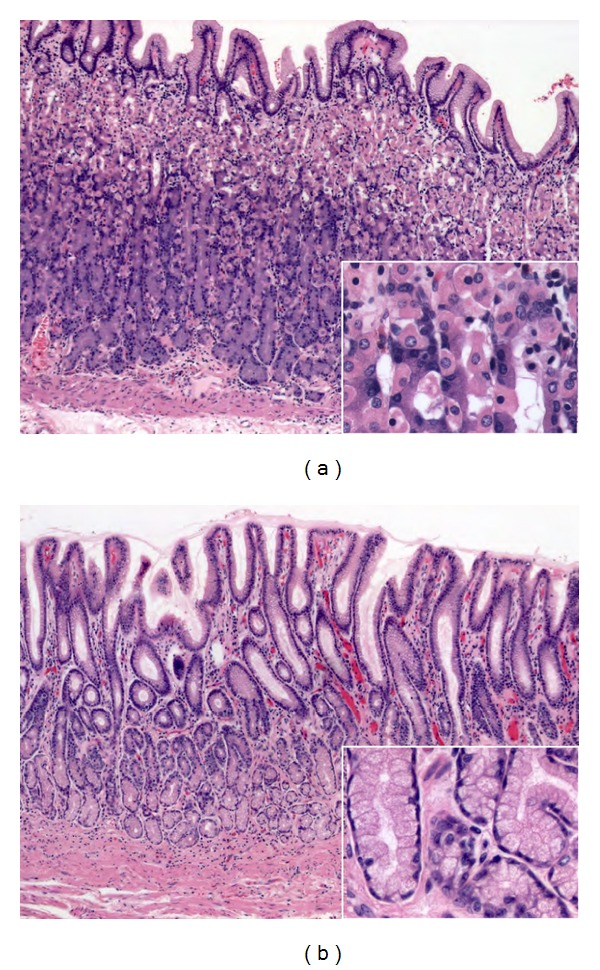
Normal gastric mucosa histology. ((a); H&E 10x; square 40x) Fundic glands are simple, branched tubular glands that extend from the bottom of the gastric pits to the muscularis mucosae; the more distinctive cells are parietal cells. ((b); H&E 10x; square 40x) Antral mucosa is formed by branched coiled tubular glands lined by secretory cells similar in appearance to the surface mucus cells.

1. Cardiac glands are limited to a narrow region of the stomach (the cardia) that surrounds the esophageal orifice. They are tubular, somewhat tortuous, and occasionally branched, and are mainly formed by mucus-secreting cells, with occasional interspersed enteroendocrin cells.
2. Fundic glands are present throughout the entire gastric mucosa, except for relative small regions occupied by cardiac and antral-pyloric glands. The fundic glands are simple, branched tubular glands that extend from the bottom of the gastric pits to the muscularis mucosae (Figure 1), and are formed by four functional types of cells: mucous neck cells, chief cells, enteroendocrine cells, parietal cells (also called oxyntic cells), and undifferentiated cells.
3. Antral-pyloric glands are located in the *pylori* antrum (the part of the stomach between the fundus and the pylorus). They are branched coiled tubular glands and are lined by secretory cells similar in appearance to the surface mucus cells (Figure 1), suggesting a relative viscous secretion. Enteroendocrine cells are found interspersed within the gland epithelium along with occasional parietal cells.

The lamina propria is relatively scant and restricted to the limited spaces surrounding the gastric pits and glands. The stroma is composed of reticular fibers with associated fibroblasts and smooth muscle cells. It is also composed of lymphocytes, plasma cells, macrophages, and some eosinophils. The lymphocytes are predominantly immunoglobulin (Ig) A-producing B cells. IgG- and IgM-secreting cells are also present. Under normal conditions, intraepithelial lymphocytes are not present anywhere in the gastric mucosa. There is also a small number of lamina propria T cells, neutrophils, and mast cells. The lamina propria also contains capillaries, arterioles, and nonmyelinated nerve fibers. Small lymphoid aggregates, usually located in close proximity to the muscularis mucosae at the base of the lamina propria, especially at the corpus, could be present in normal gastric mucosa. In contrast, the presence of lymphoid aggregates with germinal centers is extremely rare in the mucosa of normal *H. pylori* negative adults [5].

## 3. Histopathology of *Helicobacter pylori*-Induced Chronic Gastritis 

*H. pylori* is a microaerophilic gram-negative bacteria. The early phase of *H. pylori* infection elicits an acute inflammatory response that is either asymptomatic, or symptomatic with short-lived clinical manifestations such as nausea and vomiting, that evolve to a long-standing chronic gastritis [4]. Its prevalence goes from less than 15% in some populations to virtually 100%, depending on socioeconomic status and country development [6]. In industrialized countries (Western Europe, United States, Canada, and Australia), exposure tends to occur later in life, which results in a lower percentage of infected adults. An average of 20% to 30% of adults is infected by age 50 [6] and the prevalence of *H. pylori* infection has been steadily declining in emerging countries, which is probably a reflection of improved sanitary conditions, as well as the widespread use of antibiotics. The foveolar epithelium produces a thick layer of mucus that plays a protective role. This mucus layer is the primary site for *H. pylori* colonization [7]; so, they characteristically attach to the surface mucous cells, but do not penetrate them. In chronic infection, *H. pylori* contacts the surface epithelial membrane, producing prominent epithelial degeneration [8, 9]. The cells often become irregular and cuboidal in shape, showing a decrease in apical mucin content, as well as occasional “drop outs,” which leaves small gaps in the epithelium and contributes to a ragged, disorderly appearance.

**Figure 2:**
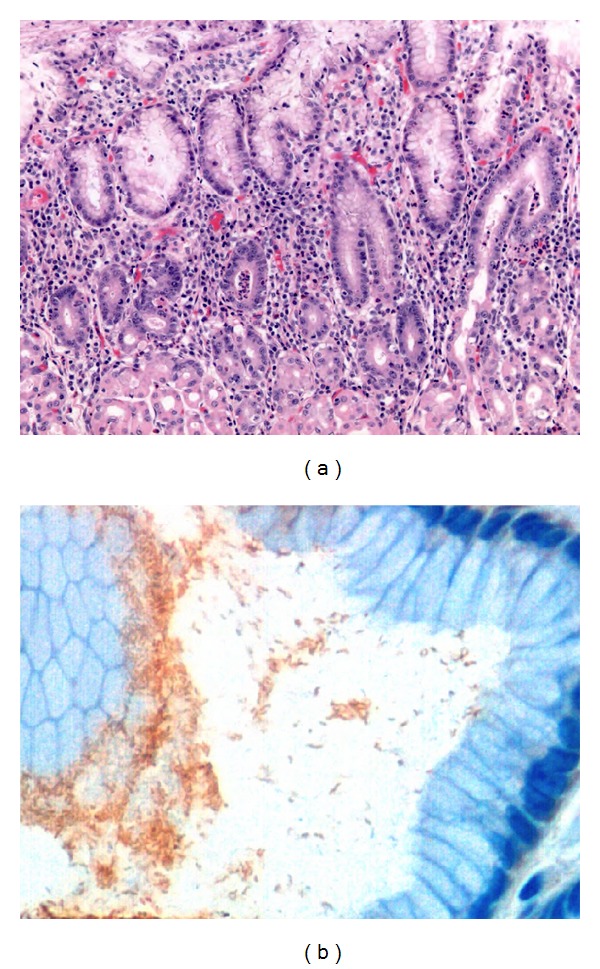
Early acute superficial gastritis. ((a); H&E 20x) Marked neutrophilic infiltrates appear in the mucous neck region and lamina with a pit micoabscess. ((b); *H. pylori* immunostaining: rabbit polyclonal; clone CH-20 429, Novocastra; 40x) Numerous *H. pylori* bacteria are present in the superficial foveolar epithelium.

*H. pylori* preferentially colonize the antrum, but they may infect any part of the stomach where it causes gastritis. When treated, the bacteria migrate from the antrum to the corpus, decreasing the activity of antral gastritis. Marked neutrophilic infiltrates appear in the mucous neck region and lamina propria in early acute gastritis (Figure 2); when severe, they aggregate in the pit lumens to form pit abscesses. Both the neutrophils and the *H. pylori* destroy the epithelium, causing the mucous neck cells to proliferate in an effort to replace the dying cells. The regenerative pit bases are characterized by mucin loss, cytoplasmic basophilia, increased mitoses, and hyperchromatic nuclei that are sometimes severe enough to mimic dysplasia.

Neutrophilic inflammation and the presence of lymphoid follicles with germinal centers are the two most distinctive histological features of *H. pylori* infection, and its eradication causes rapid neutrophil disappearance; thus their continued presence is considered a valuable indicator of therapeutic failure. The surface changes reverse rapidly, and the epithelial cells acquire their normal shape and spatial organization within a few days of *H. pylori* eradication. However, any atrophy that had developed remains, as did the lymphoid aggregates [10]. These features become permanent components of the once-infected gastric mucosa.

## 4. Grading the *Helicobacter pylori*-Induced Chronic Gastritis: The Updated Sydney System

**Figure 3:**
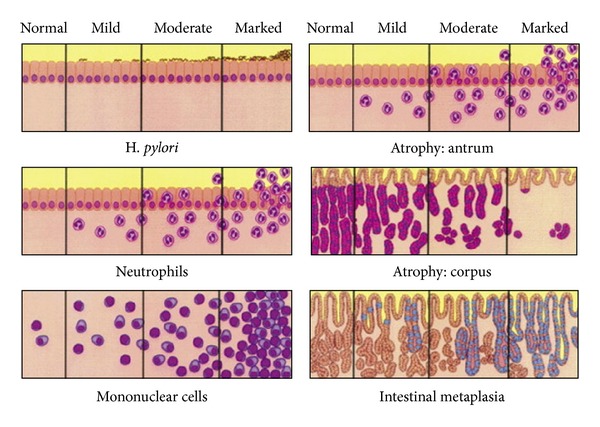
The Updated Sydney System visual standardized visual analogue scale. Each feature is assigned either a numeric or descriptive value: 0 for absent, 1 for mild, 2 for moderate, and 3 for marked (or severe). Taken from Dixon et al. [11].

The most widely used grading system for gastritis is the Updated Sydney System [11]. The system provides guidelines for generating systematic and uniform diagnostic reports. The goal of the Sydney System is to make gastric biopsy pathology reporting consistent, so that clinical studies can be performed and evaluated in a meaningful manner. The system classifies chronic gastritis on the basis of topography, morphology, and, when possible, etiology, into three broad categories: acute, chronic, and special (or distinctive). The biopsy protocol recommends that specimens from three compartments (i.e., antrum, incisura angularis, and corpus) should be separately designated when submitted to the pathology laboratory. Each relevant pathologic feature (density of *H. pylori*, intensity of neutrophilic and mononuclear inflammation, atrophy of the antrum and corpus, and intestinal metaplasia) should be graded on a standardized visual analogue scale (Figure 3). Each feature is assigned either a numeric or descriptive value: 0 for absent, 1 for mild, 2 for moderate, and 3 for marked (or severe). The values of each specimen are then averaged separately for each anatomic compartment (antrum and corpus). The next step is to document the degree of inflammation in the two main gastric compartments (antrum and corpus) and to determine whether the inflammation is similar in intensity (i.e., pangastritis) or more severe in either the antrum (antrum-predominant gastritis) or the corpus (corpus-predominant gastritis).

## 5. *Helicobacter pylori*-Induced Chronic Gastritis and Multistep Cascade of Gastric Carcinogenesis

**Figure 4:**
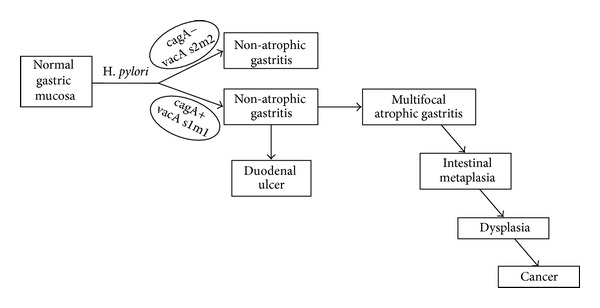
Multistep cascade of gastric cancer. This sequence begins with the infection of *H. pylori* to sequential steps of the precancerous cascades. Taken from Correa and Piazuelo [20].

In gastric cancer development, *H. pylori*-induced chronic gastritis is the first step of the so-called multistep cascade of gastric cancer. The cascade sequence of gastric carcinogenesis includes the nonatrophic chronic gastritis, multifocal atrophic gastritis, intestinal metaplasia, low-grade dysplasia (low-grade noninvasive neoplasia), high-grade dysplasia (high-grade noninvasive neoplasia), and invasive adenocarcinoma as described by Correa as the “human model of gastric carcinogenesis” [12]. This multistep model hypothesizes that the sequence of lesions reflects a dynamic process from a naïve inflammation caused by *H. pylori* infection to a fully malignant neoplasm of the stomach [13–16]. Independent epidemiological studies have confirmed that these entities are all linked through a sequential cause-effect relationship, thus supporting the concept of a human model for gastric carcinogenesis [17–19]. In a recent review and update of this model, it is also postulated that *H. pylori* is present not only in the first step of gastric mucosa inflammation but as an etiological factor in every step of the precancerous cascade [20]. In the first step, *H. pylori* infection targets normal mucosa with well-preserved gastric glands, by definition such gastritis is nonatrophic. At this point, it can be cured by clearing *H. pylori* infection or it may be evolving in two ways: it can remain as nonatrophic or it progresses in severity, leading to damage to the gastric glands, which may eventually disappear [20]. The progression depends on the interplay of three sets of etiological factors: infectious agent, host's genetic susceptibility and external environment; these determine the susceptibility and severity of outcome in the subset of individuals that develop clinical disease [20, 21]. The presence of virulent factors in the infecting *H. pylori* strain is a known determinant factor of the outcome of the infection. Infection with cag-positive vacA s1m1 strains is associated with the development of gastric cancer, while cag-negative vacA s2m2 infection does not increase the risk of cancer and is associated to the persistency of nonatrophic gastritis (Figure 4) [20, 22].

## 6. Assessing Risks for Gastric Cancer: The Operative Link for Gastritis Assessment (OLGA)

**Figure 5:**
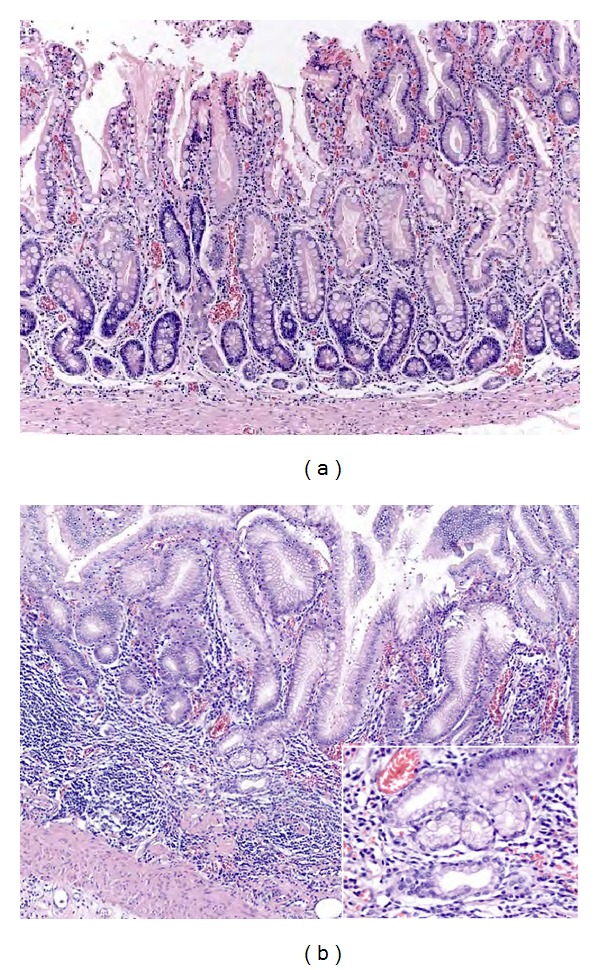
Atrophy is the loss of appropriate glands. ((a); H&E 10x) Antral gastric mucosa with accentuated atrophy because replacement by extensive intestinal metaplasia. ((b); H&E 10x; square 20x) Fundic-corporal gastric mucosa with extensive loss of gastric glands, partially replaced by pseudo-pyloric metaplasia.

The risk of malignant transformation of the lesions associated with the multistep cascade of gastric cancer is poorly defined. Long-term follow-up studies have shown a risk from 10% to 17% in the case of dysplasia [23–27]. For intestinal metaplasia, the risk assessment has conflicting results and therefore a limited clinical value [28–32]. The recently developed Operative Link for Gastritis Assessment (OLGA) staging system [33], through the evaluation of the extension and site of the atrophic changes, is an attempt to evaluate the risk of chronic gastritis to progress to intestinal metaplasia and gastric cancer [34–36]. Long standing *H. pylori* infection may lead to the loss of functional glands (atrophy) and replacement of the normal gland and foveolar epithelium by intestinal type cells (intestinal metaplasia) (Figure 5), the two main histological abnormalities invariable present as the background of gastric cancer [37]. The extent and site of the atrophic changes significantly correlate with cancer risk [38]. Two main types of atrophy can be recognized: one characterized by the loss of glands, accompanied by fibrosis or fibromuscular proliferation in the lamina propria, and the other characterized by the replacement of the normal (native) glands with metaplastic glands (i.e., glands that do not normally belong to that area) [39]. The degree of atrophy and metaplasia can be assessed with OLGA staging system for atrophy risk assessment [33]. This new system requires that specimens are taken according to the biopsy protocol of the Sydney System, and that atrophy is scored in a four-tiered scale (0–3) according to the visual analogue scale of the Houston-updated Sydney system. The stage resulting from the combination of atrophic changes was assessed in the two mucosal compartments considered herein (Table 1).

## 7. Assessing Risks for Gastric Cancer: The Epigenetic Bases of Chronic Gastritis

**Figure 6:**
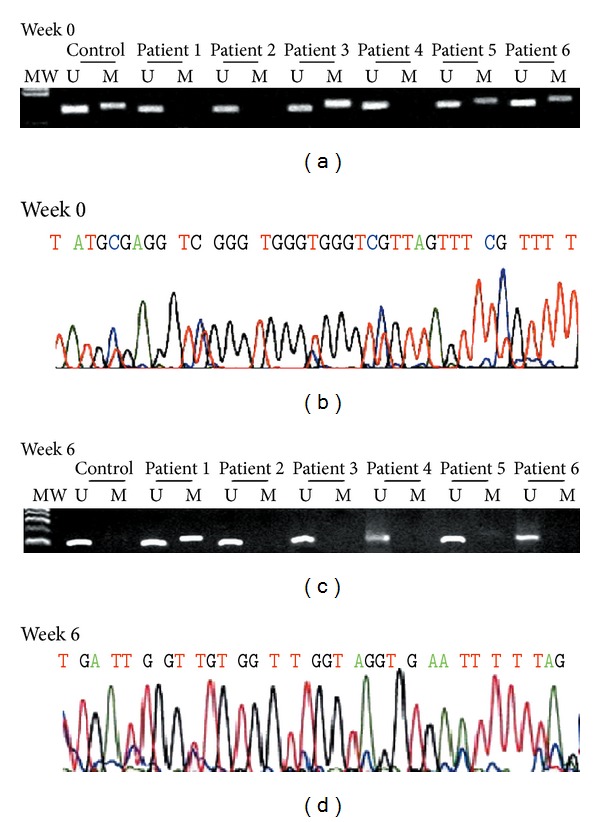
CpG island methylation pattern at the E-cadherin gene in gastric mucosa from patients with dyspepsia. (a) Before eradication of *H. pylori* (week 0), methylation was present in patients 3, 5, and 6. (b) The methylated product was confirmed by sequencing using the same methylated primer. (c) After eradication of *H. pylori* (week 6), methylation was not present in any patient. (d) The methylated product was again confirmed by sequencing using the same methylated primer. No methylated cytosine was seen. MW: molecular weight marker, U: unmethylated band, M: methylated band, red color: unmethylated cytosines converted to thymidine, blue color: methylated cytosines. Taken from Chan and Rashid [45].

**Figure 7:**
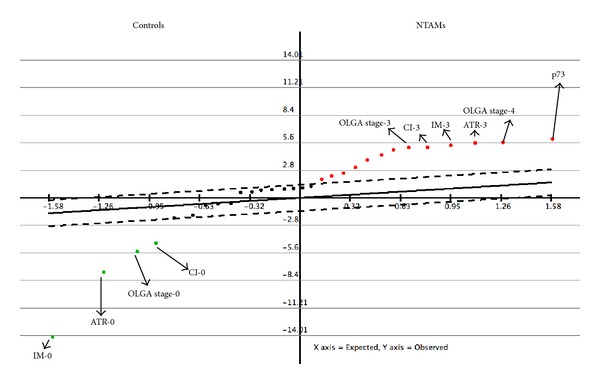
Serial analysis for microarray from nontumor adjacent mucosa (NTAM) and chronic gastritis controls. NTAM group is significantly characterized by the overexpression of p73, OLGA stages III to IV, and severe atrophy (ATR-3), intestinal metaplasia (IM-3), and chronic inflammation (CI-3) according to the Sydney System. Control group cases were significantly characterized by lack of intestinal metaplasia (IM-0), atrophy (ATR-0), and chronic inflammation (CI-0). False discovery rate = 0. Taken from Carrasco et al., [51].

The molecular bases of the multistep process of gastric carcinogenesis are highly relevant since it contributes greatly to assess risks of gastric cancer. Therefore, chronic gastritis should be understood as a disturbance in the balance between tumor suppressor genes and oncogenes. Many tumor suppressor genes have been identified in gastric cancer as well as chronic gastritis [40]. For example, inactivation of p53 tumor suppressor gene, E-cadherin, and DNA mismatch repair genes (hMSH2 and hMLH1) responsible for loss of heterozygosity (LOH) and microsatellite instability (MSI) are well-recognized examples. However, multiple studies have shown that mutation and/or deletion is an infrequent mechanism of inactivating these well-established tumor suppressor genes [41]. In this scenario, epigenetic alterations, such as DNA methylation, have been proposed as an alternative mechanism for inactivation of tumor suppressor genes [41]. DNA methylation is a process in which cytosines acquire a methyl group in 5′ position only if they are followed by a guanine (CpG site) [42]. Since DNA methylation has been considered as an excellent candidate to explain how environmental factors may increase the risk of cancer, it has been proposed as a key element for the early events of gastric carcinogenesis [43]. For example, Chan et al. [44] found that DNA methylation of the promoter region of E-cadherin has been associated with *H. pylori* infection. This association was independent of the age and/or type of gastritis [44]. Furthermore, the same authors [45] also demonstrate that *H. pylori* eradication with antibiotics reverses the DNA methylation of E-cadherin (Figure 6). Similarly, Maekita et al. [46] analyzed the effect of *H. pylori* infection on DNA methylation for multiple genes (HAND1, HRASLS, LOX, p16, P41ARC, and THBD) in *H. pylori* negative and positive healthy donors and gastric cancer patients. Among healthy donors, methylation levels were higher in *H. pylori* positives than in *H. pylori* negatives [46]. Taken together these data suggest that *H. pylori* infection induces DNA methylation mostly in premalignant conditions rather than gastric cancer itself [46]. Among other tumor suppressor genes inactivated by DNA methylation, Reprimo (RPRM), a downstream mediator of p53-induced G2 cell cycle arrest [47], has been recently associated with more aggressive *H. pylori* strains (cag-positive, vacA s1 m1, and EPIYA polymorphisms) in Colombian residents from areas with high incidence of gastric cancer [48]. Furthermore, we have identified that DNA methylation of RPRM is not only found in gastric mucosa but also in the plasma of gastric cancer patients [49]. Therefore, this circulating cell-free DNA offers the opportunity for noninvasive assessing risks for gastric cancer. Since a recent meta-analysis suggests that among several candidates, circulating cell-free DNA of RPRM methylation is the most promising [50], this issue is currently under evaluation (Detection of Methylated Reprimo in Plasma for Asymptomatic Gastric Cancer, DEMRAC study). Finally, we have recently attempted to identify specific tissue biomarkers for assessing the risk of premalignant gastritis [51]. To this purpose, we evaluate the tissue overexpression, as a consequence of DNA hypomethylation, of several oncoproteins associated with gastric cancer including STAT1, p73, FHIT, p16INK4a, BRCA1, HSP90, and EGFR. These tissue biomarkers were compared with *H. pylori* and the OLGA staging system. As expected, severe atrophy and OLGA stage IV were the most relevant histological features of premalignant gastritis. Among tissue biomarkers, overexpression of p73 was the most relevant finding. Both data were integrated by systems pathology approach by performing Significance Analysis of Microarrays [52, 53]. This approach shows that p73 is stronger as a single variable when compared with OLGA stage IV (Figure 7). Therefore, we believe that gastritis with severe atrophy has a greater risk for developing gastric cancer if, in addition, overexpress p73 [51].

In summary the role of *H. pylori* in the development of chronic gastritis is not only associated to the bacteria itself but also to host and environmental factors. Assessing risks for gastric cancer can be achieved by the evaluation of clinical, morphological, and molecular factors. Among morphological criteria, atrophy and intestinal metaplasia can be evaluated via Sydney and OLGA approaches. Molecular factors that should be considered are mostly E-cadherin methylation, circulating cell-free DNA of RPRM methylation, and overexpression of p73. Finally, a system pathology approach allows integrating all these factors that might be useful in switching from an interpretive and subjective morphologically oriented approach to a more objective, evidence-based tissue marker approach.

## Figures and Tables

**Table 1:** The OLGA staging frame. Atrophy is scored as the percentage of atrophic glands and scored on a four-tiered scale. No atrophy (0%) = score 0; mild atrophy (1–30%) = score 1; moderate atrophy (31–60%) = score 2; 9 severe atrophy (>60%) = score 3. These scores (0–3) are used in the OLGA staging assessment in each 10 compartment. Taken from Rugge et al., Dig Liver Dis 2011; 43:S373-84 with permission of Elsevier.

		Corpus			
	Atrophy score	No atrophy	Mild atrophy	Moderate atrophy	Severe atrophy
		(score 0)	(score 1)	(score 2)	(score 3)
Antrum	No atrophy (score 0) (including incisura angularis)	Stage 0	Stage I	Stage II	Stage II
	Mild atrophy (score 1) (including incisura angularis)	Stage I	Stage I	Stage II	Stage III
	Moderate atrophy (score 2) (including incisura angularis)	Stage II	Stage II	Stage III	Stage IV
	Severe atrophy (score 3) (including incisura angularis)	Stage III	Stage III	Stage IV	Stage IV
